# The Expression of Neurodegeneration-Related Genes in the Hippocampus of Hypothyroid Rats Following Long-Term Potentiation

**DOI:** 10.5152/eurasianjmed.2024.23133

**Published:** 2024-02-01

**Authors:** Melek Altunkaya, Ercan Babur, Özlem Barutcu, Cem Süer, Nurcan Dursun

**Affiliations:** 1 Department of Medical Services and Techniques Services, Selçuk University Vocational School of Health Services, Konya, Turkey; 2 Department of Physiology, Erciyes University Faculty of Medicine, Kayseri, Turkey

**Keywords:** Gene expression, hippocampus, hypothyroidism, neuroplasticity

## Abstract

**Background:**

In our research, we examined how the induction of long-term potentiation (LTP) in the hippocampus of hypothyroid rats affects the mRNA levels of several proteins involved with neurodegeneration, including Gsk3, Cdk5, Akt1, Mapt, P35 (Anxa), Capn1, Bace1, and Psen2.

**Methods:**

Wistar-albino rats, consisting of 12 males, were used in the research, and they were separated into 2 groups: control (n = 6) and hypothyroidism (n = 6). To induce hypothyroidism, propylthiouracil was added to drinking water at a dosage of 20 mg/kg/day. The test stimulus intensity was calculated, basal recordings were acquired, and LTP was induced by administering 100 Hz high-frequency stimulation (HFS) for 1 second with a 5-minute delay when the rats were aged 60 days. The population spike (PS) amplitude and excitatory postsynaptic potential (EPSP) slope were measured in the granule cell layer of the dentate gyrus. Using reverse transcription polymerase chain reaction, the mRNA levels of neurodegenerative genes were assessed in induced hippocampal tissues after the LTP protocol. The free T4 levels in plasma were measured using a plate reader with the commercial ELISA kit.

**Results:**

Following HFS, LTP was solely induced in the EPSP slope and PS amplitude in the control group. The impaired LTP response of the hypothyroidism group was accompanied by an increase in Akt1-mRNA expression and a decrease in Gsk3ß expression, whereas the value genes’ mRNA expression levels did not differ significantly from those of the control group.

**Conclusion:**

The hypothyroidism-related LTP impairment could be caused by a reduction in PI3K/AKT signaling**. **Further investigation of this path is required to elucidate the pathophysiology of impaired synaptic plasticity in hypothyroidism.

## Introduction

Thyroxine (T4) and triiodothyronine (T3), 2 thyroid hormones, are crucial for brain growth and control fundamental metabolic processes that have an impact on nearly all human organ systems. Hypothyroidism is associated with insufficient levels of these chemicals.^1,2^ Thyroid hormones either bind to their nuclear receptors and activate transcriptional activation, or they activate the Pi3k-Akt/Gsk3ß and mitogen-activated protein kinase (MAPK) signaling pathways through the cell-surface protein known as the avß3 integrin receptor.^3-5^

The type of synaptic plasticity that has lately received attention is LTP.^6^ Increased frequency of stimulation between neurons leads to the quality of synaptic connections.^7,8^ *N*-methyl-d-aspartate (NMDA) postsynaptic receptor stimulation initiates LTP.^9^ Here, the type of plasticity that might occur depends on the rates of Ca entrance into the NMDA receptor (fast input, delayed input). A condition known as long-term depression—the weakening or removal of synapses—occurs when there is a slow and prolonged Ca input. Long-term potentiation indicates the growth and greater efficacy of synapses resulting from the activation of kinases under conditions of fast and short-term Ca input. It consists of 2 phases. While late LTP necessitates the synthesis of mRNA and proteins, early LTP includes the modification of proteins independent of gene expression and protein synthesis. In addition to CaMKII, which is considered to be required for LTP, it has been discovered that a number of proteins, such as calpains, another type of Ca^+2^-dependent enzyme PKA, various versions of PKC, MAPK, and tyrosine kinases, contribute to LTP. The main function of these signaling pathways in LTP is highly complicated and changes depending on the stage of the process.^9-12^ NR2b and NR1, the subunits of NMDA receptor, which are crucial for the development of LTP, are regulated by thyroid hormones.^13^ The fact that NMDA receptors are an essential part of synapses that play a role in synaptic plasticity, memory, and learning processes emphasizes how crucial thyroid function is for maintaining healthy neural communication.

The activity of numerous kinases and phosphatases can be altered by thyroid hormone, which acts on both the nucleus and the membrane. We believe that the abnormal activity of these kinases and phosphatases, which function as signaling networks, may contribute to neurodegenerative disorders. Therefore, the goal of our research was to examine the changes in the mRNA levels of Cdk5, Akt1, Mapt, GSK3, P35, Capn1, Bace1, and PSEN2 that followed the induction of plasticity and were related to neurodegenerative proteins in the hippocampus of hypothyroid rats.

## Material and Methods

This research was completed as part of the TDK-2019-9405 project, which was funded by the University Scientific Research Projects (BAP) Unit. It also received the 19/133 clearance of the Erciyes University Animal Experiments ethics committee, which was issued on July 17, 2019. In the study, ethical principles were seen as crucial to avoid using experimental animals unnecessarily and inflicting pain. Our study included 12 male Wistar albino rats, each weighing between 180 and 200 g, housed at the Experimental and Clinical Research Institute at the University.

### Inducing Hypothyroidism

Rats were given 20 mg/kg/day of propylthiouracil (Sigma; inventory number: P3755) for 21 days beginning on day 39 in order to induce hypothyroidism.^14^ Six rats from the control group were given regular tap water.

### Electrophysiological Recording

. Rats were anesthetized with urethane (1.2 g/kg), and then placed in stereotaxis using ear and mouth sticks. Taking Bregma as a reference point, a two-channel glass micropipette filled with 3 M NaCl was lowered down to anteroposterior (AP):6.5 and mediolateral (ML):3.8 mm coordinates of stimulating electrode, and the AP: 3 mm ML: 2.13 mm coordinates of recording electrode in the hippocampus until observing the typical response. The input/output studies were used to determine the test stimulus intensity, which was equal to half of the highest intensity received from the dentate gyrus (DG) granule cells through the recording electrode and to conduct the basal recording for 15 minutes on the perforant pathway (PFP). Field potentials in the DG granule cell layer were recorded for 15 minutes, while PS amplitude and EPSP slope were measured during basal recording by stimulating the PFP with the predetermined stimulus intensity every 30 seconds. After basal recording, LTP was initiated using HFS that lasted 1 second at a frequency of 100 Hz and an interval of 5 minutes (a total of 15 minutes). After the HFS, the stimulation was continued by adding a test stimulus every 30 seconds for another 60 minutes while recording the EPSP slope and PS amplitude.

### Measurement of Plasma-Free T4 Levels

The free T4 levels in plasma were measured using a plate reader (MultiskanTM FC Microplate Photometer) with the commercial ELISA kit (Cloud Cone Corporation, San Fransisco, California, USA). The sensitivity of the radioimmunoassay for free T4 was 0.0023 ng/dL. Readings under the limit of detection were recorded at 0.023 ng/dL for statistical purposes. All samples were done in duplicate.

### Reverse Transcription Polymerase Chain Reaction Measurements

Following electrophysiological measurements, rats were decapitated, and hippocampus tissues were removed. All tissues were stored at −80°C, and the polymerase chain reaction (PCR) study was started after the hippocampus tissue of all rats was collected (2-weeks electrophysiological recording period). In the hippocampal tissue, the mRNA levels of proteins associated with neurodegeneration (Gsk3ß, Cdk5, Akt1 P35 (Anxa), Calpn1, Mapt, Bace1, and Psen2) were assessed.

### RNA Isolation

Using a homogenizer, the hippocampal tissues were combined with 1000 µL of RNA extraction solution (PureZOLTM: BioRad, Hercules, California, USA). Then 400 µL of chloroform was added, and a 15-second vortex was performed. They were kept for 15 minutes at room temperature before being centrifuged at 14 000 g for 20 minutes at +4°C. A new Eppendorf tube was used to contain the aqueous phase that had developed at the end of the centrifugation. Depending on the volume of the aqueous phase, 300 µL of isopropanol was added, and the container was repeatedly turned upside down. They were centrifuged for 30 minutes after being kept at room temperature for 10 minutes. At the end of the centrifugation, the supernatant portion was discarded. Before vortexing, 1 mL of 75% ethanol was added to the particle that was still at the bottom. It was then centrifuged for 5 minutes at +4°C at 7500 g. After discarding the supernatant part created after centrifugation, ethanol surrounding the pellet was removed with a pipette. The particle was dried for 10-15 minutes at room temperature, allowing the ethanol to evaporate. Twenty microliter of nuclease-free water (NFW)was added after the drying procedure, and the particle was resuspended. It was covered with ice and stored at +4°C for 10-15 minutes. RNA concentration (ng/µL) was measured in the NanoDrop at the end of this procedure.

### cDNA Synthesis

According to the iScripTM procedure, the iScrip TMcDNA synthesis Kit was used to synthesize cDNA (1708890, BioRad). To establish a reaction with an equal amount of RNA from each sample, the amounts of RNA and water that needed to be supplied were decided upon in accordance with the findings of the RNA. The PCR tubes with a total capacity of 15 µL were filled with the required number of NFW and RNA samples. Then, each PCR tube was filled with 5 µL of a combination of 5× reaction buffer (4 µL) and reverse transcriptase (1 µL), and the tubes were put in a PCR device. At the end of the PCR, we obtained 20 µL of cDNA output. For the incubation, denaturation, proliferation, and fusion procedures, the product was kept at 25°C for 5 minutes, 42°C for 30 minutes, and 85°C for 5 minutes in the PCR device (CFX Connect Real-Time PCR Detection System, BioRad).

### mRNA Expression

Using the SsO Advanced Universal IT SYBR Green Supermix reagent (10000076382, BioRad, USA) in the BIORAD CFX Connect Real Time PCR device, the mRNA expressions of the genes Akt1, Cdk5, Gsk3ß, Mapt, P35, Bace1, Calpn1, and Psen2 were examined. One microliter of cDNA was added to the plate after a reaction mixture consisting of 10 µL SYBR Green Supermix, 8 µL Nuclear Free Water, 0.5 µL Primer F, and 0.5 µL Primer R. Then, it was placed in the CFX Connect Real-Time PCR detection System. For preincubation, replication, and cooling, the PCR was kept at 95°C for 3 minutes, 5°C at 95°C, 15°C at 60°C (45 cycles), and 30°C at 40°C, respectively. In order to avoid manipulation errors, all samples were examined in duplicates. The housekeeping gene was the β-actin gene. It was then calculated and normalized using the 2^−Δ^ *^Ct^* (threshold cycle) value method.

### Statistics Analysis

According to calculations, the slope of the EPSP waveform was 80%-20% of the voltage differential between the start of the wave and the start of the PS wave. The average of the initial negative altitude and the following positive altitude was used to determine the PS amplitude. The average EPSP slope and PS amplitude values of the 30 field potentials triggered during the initial 15-minute basal recording were considered to be 100. The 10 field potentials that were collected during the first 5 minutes following HFS and the final 5 minutes of the experiment were used to calculate the averages of the PS amplitude and EPSP slope values.

The values found within a group and the basal values were compared using the single-sample *t*-test. The significance between the groups was assessed using the Mann–Whitney *U*-test. Using histograms, *q*–*q* graphs, and the Shapiro–Wilk test, the conformity of the data acquired through real-time PCR to the normal distribution was assessed. Using Levene’s test, the homogeneity of variation was examined. The program GraphPad Prism was used to analyze the data. Real-time PCR and sT4 level measurement results were analyzed using the Mann–Whitney *U*-test to determine whether there were any significant differences between the groups. In each experiment, the significance level was set at *P* = .05.

## Results

### Plasma-Free T_4_ Level

The group given propylthiouracil had lower plasma fT4 levels compared to the control group (control group: 35.0 ± 8.4 pg/mL; hypothyroidgroup: 21.0 ± 8.5 pg/mL; *P* < .02). This result indicated that hypothyroidism developed in rats.

### Analysis of Input/Output Curves

**Figure 1.**
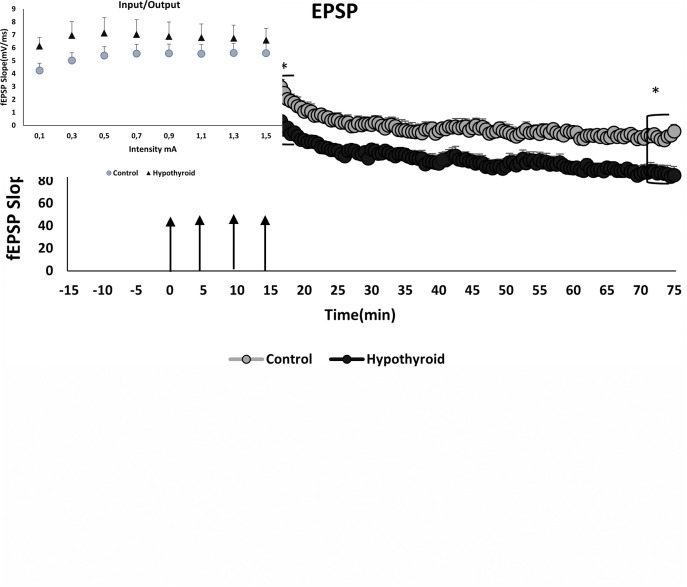
Excitatory postsynaptic slopes. (A) EPSP slopes recorded during the experiment. (B) EPSP slopes in the post-high-frequency stimulation period. (C) EPSP slopes in the maintenance period. (D) Input/output curves of the EPSP slopes. It represents the significant increase according to the basal value. EPSP, excitatory postsynaptic potential. * represents a significant difference compared to the control group (n = 6) (*P* < .004).

**Figure 2.**
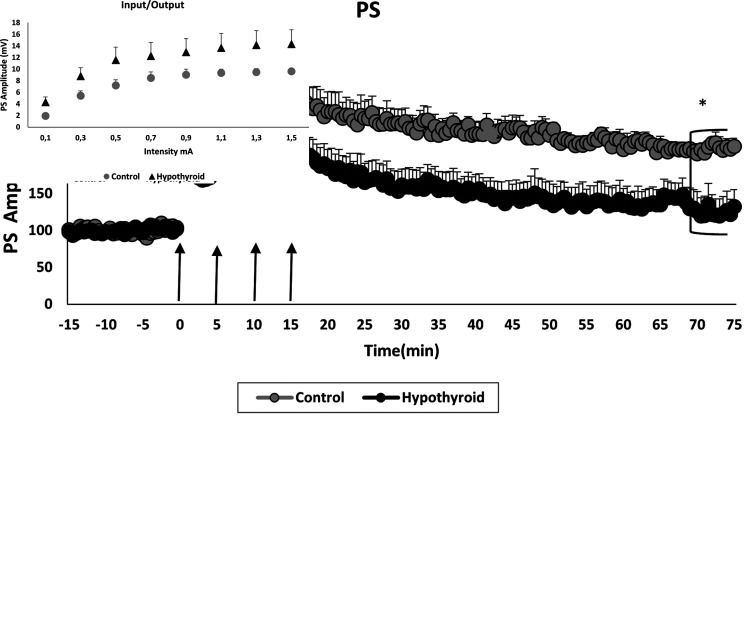
Population spike amplitudes. (A) PS amplitude recorded during the experiment. (B) PS amplitudes in the post-HF period. (C) PS amplitudes in the maintenance period. (D) Input/output curves of the PS amplitudes. It represents the significant increase according to the basal value. PS, population spike. * represents a significant difference compared to the control group (n = 6) (*P* < .01).

Using input–output curves, the impact of propylthiouracil administration on synapses’ and neurons’ basal activity prior to plasticity activation was assessed. Graphics were used to display the average and standard error values of the EPSP slope and PS amplitude recorded in reaction to stimulus intensity (Figure 1D; Figure 2D).

**Figure 3.**
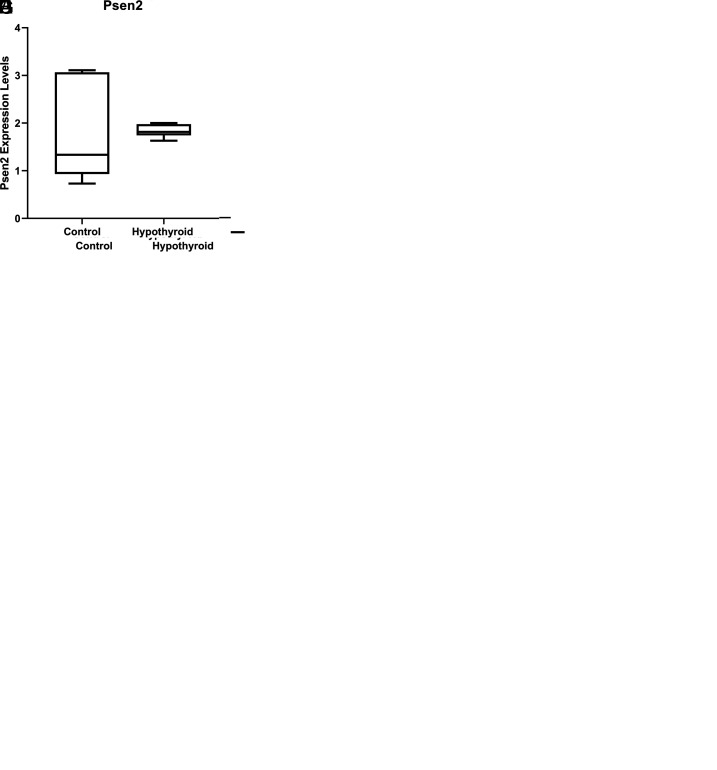
mRNA expression levels of neurodegenerative protein-related genes (n = 6). (A) Gsk3β. (B) Akt1. (C) Cdk5. (D) Mapt. (E) Anxa. (F) Capn1. (G) Bace1. (H) Psen2. The data are expressed as median (first quarter-third quarter). * represents a significant difference compared to the control group.

Before HFS, input/output curves were measured by applying pulses of intensity in the range of 0.1-1.5 mA. The results showed that there was a significant increase in EPSP slopes (*F*_7.70_ = 5.49; *P* < .001 Figure 1D) and PS amplitudes (*F*_7.70_ = 29.69; *P* < .001 Figure 3D) as the intensity of the pulses increased. The fact that the interaction between EPSP slope (*F*_7.70_ = 1.31; *P* = .29) and PS amplitude (*F*_7.70_ = 0.49; *P* = .60 following the lower-bound correction) values and stimulus intensity was not significant showed that this increase occurred similarly in all groups. The group effect for EPSP slope (*F*_1.10_ = 1.50; *P* = .24) and PS amplitude (*F*_1.10_ = 2.65; *P* = .08) was not significant. These results showed that both the control and hypothyroidism groups had similar levels of activity in the perforant pathway-dentate gyrus synapses prior to the induction of LTP with HFS.

### High-Frequency Stimulation’s Impact on Excitatory Postsynaptic Potential and Population Spike

Single-sample *t*-test analyses showed that the post-HFS EPSP slopes for both the control (154.21.0%, *t*_5_ = 8.26, *P* = .00; Figure 1B) and hypothyroidism (124.111.4%, *t*_5_ = 5.17, *P* = .004; Figure 1B) groups were statistically significantly higher than those obtained in the basal phase. The maintenance period EPSP slopes (119.39.0%, *t*_5_ = 5.20, *P* = .003; Figure 1C) were found to be numerically greater in the control group than during the basal period. The EPSP slopes of the maintenance phase and the basal period did not vary statistically significantly in the hypothyroidism group (86.517.75, *t*_5_ = −1.858, *P* = .122; Figure 1C). These results showed that there was no synaptic LTP reaction in the hypothyroidism group. There was a significant difference between the groups in terms of the EPSP slopes obtained in the post-HFS period (*Z* = −2.56, *P* = .01) and in the maintenance period (*Z* = −2.72, *P* = .006).

Population spike amplitude was evaluated using the single-sample *t*-test according to the basal period. The post-HFS PS amplitude of the control (271.0 ± 62.4%, *t*_5_ = 6.716, *P* = .01; Figure 2B) and hypothyroidism (196.2 ± 60.8%, *t*_5_ = 3.876, *P* = .012; Figure 2B) groups was found to be statistically significantly higher compared to the basal period. There was no significant difference in the PS amplitude between the maintenance period and the basal period in either the control or hypothyroidism groups (control: 212.3 ± 21.9%, *t*_5_ = 12.536, *P* = .00; Figure 2C; hypothyroidism: 124.2 ± 58.0, *t*_5_ = 1.005, *P* = .361; Figure 2D). There was a difference between the post-HFS PS amplitude (*Z* = −2.08, *P* = .037) and the maintenance period PS amplitude (*Z* = −2.56, *P* = .01) of the groups.

These results showed that, compared to the control group, the hypothyroidism group did not induce LTP in either the synapse or somatic components, indicating that hypothyroidism interfered with the LTP response.

### Real-Time Polymerase Chain Reaction Analysis

The mRNA expression levels of genes associated with neurodegenerative proteins in the hippocampus are shown in the graph in Figure 3.

The expression levels of the tau-related genes Gsk3ß and Akt1-mRNA were statistically different between the groups, according to the Mann Whitney U test, while the expression levels of Cdk5, P35, Capn1, Bace1, Mapt1, and Psen2-mRNA were not statistically different (*P* > .05).

## Discussion

In our study, which was carried out to understand the potential effects of hypothyroidism on gene expression, plasma-free T4 levels in propylthiouracil (PTU)treated rats decreased compared to the control group after the administration for 21 days (20 mg/kg/day dose), in accordance with both the literature and referencing doses previously administered in our laboratory.^15,16^ This situation supported the hypothesis that hypothyroidism was induced.

This procedure was adequate to induce a permanent LTP, as evidenced by the fact that the PS amplitude and EPSP slope were both noticeably higher in the control group 60 minutes after the LTP induction compared to the prior value (Figures 1 and 2). However, the procedure, which could initiate LTP in rats for at least an hour in the control group, was unable to initiate LTP in the hypothyroidism group in either the synaptic component or the somatic component. This result was consistent with the available literature and previous conclusions of our research team.^17-19^

We measured the amount of mRNA expression in our research using real-time PCR, which has a high rate of accuracy. Although the mRNA expression that we have evaluated can show variations in the sequence of gene expression and the biological process, it is not adequate to expose the function of the encoded protein. These results showed that the hypothyroidism group significantly differed from the control group in the levels of mRNA expression of the Gsk3ß and Akt1 genes after LTP recording in stimulated hippocampus tissue, whereas the levels of mRNA expression of the Cdk5, Mapt, P35 (Anxa), Capn1, Bace1, and Psen2 genes showed no significant difference between the groups.

Long-term memory development and LTP induction are both facilitated by intricate biochemical communication. This intricate communication pathway involves significant kinases. In this system and during the formation of hippocampal neurons, the important kinase GSK3ß plays a crucial role in defining the orientation of the neurons. Additionally, it is well known that stimulation of PI3K occurs during the induction of LTP, and the PI3K-Akt pathway is a crucial regulator of GSK3ß.^20^ The formation of LTP by the action of Akt1 requires Gsk3ß to remain in an inactive state.^20-22^ However, a reduction in Gsk3ß expression or a mismatch in its inactive state could impair regular functioning. In our research, the deterioration in the LTP response was attributed to an increase in Akt1-mRNA level and a decrease in Gsk3ß-mRNA level in rats with hypothyroidism.

Gsk3ß was found to be required for LTP, in addition to other proteins, including tyrosine kinases, PKA, various PKC isoforms, MAPK, and calpains, contributing to LTP.^10,23^ The lack of a significant difference between the hypothyroid and the control group in terms of the mRNA expression levels of key kinases linked to tau phosphorylation, including Cdk5, Mapt, P35 (Anxa), and Capn1, as well as amyloid precursor protein-related genes, including Bace1 and Psen2, suggested that these genes did not contribute to the impaired LTP response in both the synaptic and somatic components.

### Limitations of the Study

Limitations should be taken into account when interpreting the findings of the study: The induction of hypothyroidism was verified by the fact that plasma-free T4 levels decreased in the control group when compared to those who received PTU. We were unable to detect plasma fT3 and thyroid-stimulating hormone (TSH) levels, despite the fact that changes in fT3 and TSH levels should alter T4 levels. The real-time PCR technique used in the research only provided data on the mRNA amounts of the proteins that were tested; it did not provide data on any potential protein loss, destruction, or activity that might have happened during synthesis. Consequently, further research involving protein testing is required.

The expression of specific genes associated with plasticity induction is required to remain within physiological limits, which depends on the physiological amounts of thyroid hormones. Depending on the induction procedure, changes in hormone levels may alter how genes involved in neural plasticity are expressed at the mRNA level. However, it might suggest that GSK3ß and Akt1 can be responsible for the LTP response being compromised in thyroid hormone deficiency.
